# Prevalence of selected sexually transmitted infectious agents in a cohort of asymptomatic soldiers in Austria

**DOI:** 10.1186/s13071-022-05508-z

**Published:** 2022-11-14

**Authors:** Iwona Lesiak-Markowicz, Claudia Tscherwizek, Wolfgang Pöppl, Gerhard Mooseder, Julia Walochnik, Ursula Fürnkranz

**Affiliations:** 1 Institute of Specific Prophylaxis and Tropical Medicine, Centre for Pathophysiology, Infectiology and Immunology, Medical University of Vienna, 1090 Vienna, Austria; 2 Division of Dermatology and Tropical Medicine, Sanitätszentrum Ost, Van Swieten Kaserne, 1210 Vienna, Austria

**Keywords:** Sexually transmitted infections, Prevalence, Asymptomatic soldiers

## Abstract

**Background:**

According to the World Health Organization (WHO), more than one million sexually transmitted infections (STIs) are acquired every day worldwide. Although STIs may be asymptomatic in many cases, they can cause severe symptoms and can also lead to adverse pregnancy outcomes and both male and female infertility. Asymptomatic carriers seem to play an important role in terms of the distribution of STIs; however, studies revealing the prevalence of STIs in asymptomatic individuals are rare.

**Methods:**

In the current study, 654 leftovers of standard urine samples from healthy, asymptomatic Austrian soldiers were investigated for the prevalence of *Trichomonas vaginalis, Chlamydia trachomatis*, and genital mycoplasmas (*Mycoplasma hominis, Mycoplasma genitalium, Ureaplasma urealyticum, Ureaplasma parvum*, and *Candidatus* Mycoplasma girerdii) by specific PCRs.

**Results:**

We detected *T. vaginalis*, *M. hominis*, *U. urealyticum*, *U. parvum*, and *C. trachomatis* in the investigated samples with prevalence of 7.6%, 4%, 2.4%, 5.4%, and 3.2%, respectively; neither *M. genitalium* nor *Ca.* Mycoplasma girerdii was found in our sample collection.

**Conclusions:**

Our study introduces data on STIs of a mainly male cohort, which are scarce because most of the available information on sexually transmitted infectious agents arises from fertility clinics (mainly women) or symptomatic patients.

**Graphical abstract:**

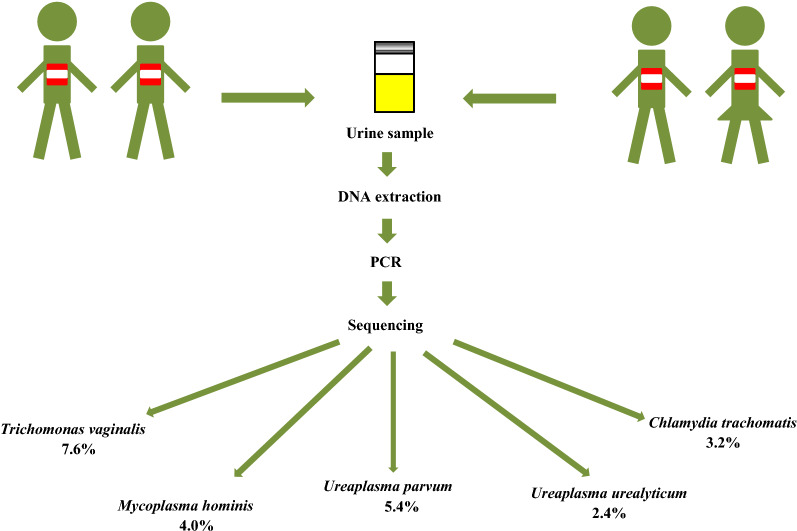

## Background

The World Health Organization (WHO) reports that approximately 376.4 million new cases of sexually transmitted infections (STIs), mainly gonorrhoea, chlamydiasis, trichomoniasis, and syphilis, are recorded worldwide every year [1]. The prevalence and incidence of STIs is influenced by many factors [2]; STIs have also always accompanied armies all over the world, mainly during deployment or in operational activities [3]. Chlamydiasis remains the most frequently diagnosed STI in soldiers; however, infections with *Trichomonas vaginalis*, *Mycoplasma hominis*, *Mycoplasma genitalium*, *Ureaplasma urealyticum*, and *Ureaplasma parvum* are also frequently observed [3, 4].

*Trichomonas vaginalis* causes trichomoniasis—the most common non-viral STI in the world. Infection with *T. vaginalis* not only can lead to female and male infertility, or preterm and/or low-weight infant births [5, 6], but can also be the cause of pelvic inflammatory disease (PID), with an increased risk of HIV transmission and acquisition [7, 8]. There is also some evidence of an association between *T. vaginalis* infection and an increased risk of cervical cancer, but more research is needed to further clarify this [9]. Around 50% of infected women and 75% of infected men remain asymptomatic; thus, men in particular frequently act as unknown carriers of *T. vaginalis*, and its chronic presence has been linked to inflammation in the prostate [10, 11].

*Mycoplasma* spp. and *Ureaplasma* spp. belong to the class Mollicutes in the family *Mycoplasmataceae* and are considered to be the smallest self-replicating organisms (0.2–0.3 µm) [12, 13]. Although genital mycoplasmas differ in shape, pathogenicity, and virulence, they are generally not considered to be the causative agents of STIs, and therefore are not included in routine screening. However, *M. hominis* is recognized as an opportunistic pathogen, which can cause genital infections, leading to endometritis and spontaneous abortion [14]. Ahmadi et al. reported that asymptomatic infection with *M. hominis* not only can negatively affect semen parameters but can also lead to male infertility [15]. *Ureaplasma urealyticum* and *U. parvum* are commensal organisms of the lower genitourinary tract of sexually active men and women; however, in some cases, ureaplasmas are strongly associated with urethritis, prostatitis, and epididymitis in men, as well as endometritis, chorioamnionitis, spontaneous abortion, and premature/low birth weight children in women. The exact role of these agents in male infertility remains a controversial subject [16]. *Ureaplasma parvum* is more likely to be a commensal, because it is detected more frequently than *U. urealyticum* ([17, 18] and own unpublished data). *Mycoplasma genitalium* is one of the major causes of non-gonococcal urethritis (NGU) worldwide, even more in men than in women [19]. There are insufficient data to associate/correlate *M. genitalium* infection with chronic conditions such as epididymitis, prostatitis, or infertility in men, and the consequences of asymptomatic infection with *M. genitalium* in men are unknown [20]. In women, infections with *M. genitalium* are often asymptomatic, but are also associated with cervicitis, PID, preterm delivery, spontaneous abortion, and infertility [20]. *Candidatus* Mycoplasma girerdii is a newly identified *Mycoplasma* species and has been detected in strong correlation with *T. vaginalis* infection [21]. A symbiotic relationship between these two microorganisms has been suggested, but *Ca.* M. girerdii has also been proven to colonize the vaginal tract alone (without *T. vaginalis* infection) [21]. *Chlamydia trachomatis* is a gram-negative obligate intracellular bacterium, infecting both men and women. Genital chlamydial infections are often asymptomatic, and can affect the upper genital tract in women, leading to PID, tubal factor infertility, ectopic pregnancy, and preterm delivery [22]. In men, epididymitis, urethral obstructions, and decreased fertility have been observed. Moreover, infection with *C. trachomatis* contributes to the spread of HIV and is associated with cervical cancer [23]. Currently, 19 serovars of *C. trachomatis* (A, B/Ba, C, D/Da, E, F, G/Ga, H, I/Ia, J, K, L1, L2, L2a, and L3) are recognized according to specific epitopes of the major outer membrane protein (MOMP). Serovars A, B, Ba, and C are known as the agents of trachoma, whereas serovars D–K are linked to oculo-genital infections, and serovars L1–L3 are associated with lymphogranuloma venereum [22]. STIs are often asymptomatic in both men and women [24, 25]; thus the infectious agents often remain undetected and are further transmitted. There are still more data on symptomatic infections than on asymptomatic infections, but we can observe that data are increasingly emerging from patients in the latter group. Treatment of asymptomatic STIs in men and women would help to control these infections [26]. The aim of the current study was to determine the prevalence of *T. vaginalis*, *M. hominis*, *U. urealyticum*, *U. parvum*, *M. genitalium*, *Ca.* M. girerdii, and *C. trachomatis* in a cohort of asymptomatic Austrian soldiers.

## Methods

### Sample collection

Leftovers of standard urine samples were collected from 654 healthy soldiers leaving for or returning from missions abroad at the Division of Dermatology and Tropical Medicine, Sanitätszentrum Ost, Van Swieten Kaserne Vienna, Austria, and sent to the Institute of Specific Prophylaxis and Tropical Medicine (ISPTM) at the Medical University of Vienna for further investigations. All samples were anonymous; no data other than gender were included in the study.

### DNA isolation

Urine samples were centrifuged at 10,000×*g* to spin down all potential bacteria and parasites. After two washing steps with phosphate-buffered saline (PBS), the QIAGEN DNA Mini Kit (QIAGEN GmbH, Hilden, Germany) was used for extraction of total DNA from the urine. The DNA was stored at −20 °C until further use. Each urine sample was tested individually; no pools were made. All samples were tested at least three times.

### *T. vaginalis* detection

For the detection of *T. vaginalis*, real-time quantitative polymerase chain reaction (qPCR) was performed. Briefly, 7.5 µl Takyon MasterMix with fluorescence dye SYBR Green was mixed with 5 µl H_2_O and 0.25 µl of TVK3 and TVK7 primers for *T. vaginalis* [27] (Table 1). Two microlitres of genomic DNA was added to a total volume of 15 µl in every slot of the qPCR assay. The qPCR was performed according to the following protocol: initial denaturation at 95 °C for 10 min, followed by 35 repeats of 15 s at 95°, 1 min at 60 °C, and 2 min at 72 °C.

**Table 1:** Primers used for the detection of *T. vaginalis, M. hominis, U. urealyticum, U. parvum, M. genitalium, Ca.* M. girerdii and *C. trachomatis*

Organism	Primer name	Primer sequence (5′→3′)	References
*T. vaginalis*	TVK3	ATTGTCGAACATTGGTCTTACCCTC	[28]
	TVK7	TCTGTGCCGTCTTCAAGTATGC	
*M. hominis*			
*U. urealyticum*	GPO-1	ACTCCTACGGGAGGCAGCAGTA	[29]
*U. parvum*	MGSO	TGCACCATCTGTCACTCTGTTAACCTC	
*Ca.* M. girerdii			
*M. genitalium*	MgPa-1	AGTTGATGAAACCTTAACCCCTTGG	[30]
	MgPa-3	CCGTTGAGGGGTTTTCCATTTTTGC	
*C. trachomatis*	momp_F	TGGAGTTAAATGGTCTCGAGC	[31]
	momp_R	GATTTCATCTTGTTCAATTGCA	

### Detection of *M. hominis*, *Ca.* M. girerdii, *U. urealyticum*, and *U. parvum*

The amplification of the *Mycoplasma*-specific 16S rRNA gene was performed with GPO-1 and MGSO primers [28] listed in Table 1 according to the protocol described by Kuppeveld et al. [28]. The amplicons were visualized on 2% agarose gels and the fragments corresponding to the expected size were cut out from the gel and purified using the Illustra™ GFX™ PCR DNA and Gel Band Purification Kit (GE Healthcare, Buckinghamshire, UK). Amplified products were sequenced with a SeqStudio (Thermo Fisher Scientific, Waltham, MA, USA) and compared with reference sequences in GenBank (www.ncbi.nlm.nih.gov/GenBank).

### Detection of *M. genitalium*

For the detection of *M. genitalium* in urine samples, *M. genitalium* DNA was amplified with specific primers [29] additionally to the procedure described for the other mycoplasmas. Amplified products were purified and sequenced as described above.

### *C. trachomatis* detection and serovar determination

Primers used for the detection of the MOMP gene (momp_F, momp_R) of *C. trachomatis* are given in Table 3. A conventional PCR was carried out following the protocol by Safarkar et al. [30]. Serovar determination was performed by *omp1* gene fragment amplification in a nested PCR as described by Hsu [31]. The final PCR products were purified from the gel and sequenced using the inner primer set. Consensus sequences were compared with sequences of reference *C. trachomatis* strains from GenBank.

## Results

### Pathogen detection in asymptomatic soldiers

We collected urine samples from 654 asymptomatic soldiers, including 15 from women 15/654 (2.3%) and 639 from men (639/654, 97.7%). We detected *T. vaginalis*, *M. hominis*, *U. urealyticum*, *U. parvum*, and *C. trachomatis* in 50, 26, 16, 35, and 21 of the samples, respectively (Table 2). *Trichomonas vaginalis* was the most common pathogen. We did not detect *M. genitalium* using mycoplasma 16S rRNA gene-specific primers, and confirmed the absence with *M. genitalium*-specific primers. We also did not detect *Ca.* M. girerdii in our samples; in six samples, “uncultured bacteria” were found (using the nucleotide basic local alignment search tool of NCBI GenBank, defined above in methods).

**Table 2:** Prevalence (number of positive/total of tested population) of identified pathogens among asymptomatic soldiers (*n* = 654)

Pathogen	Prevalence (%)	Female/Male
*T. vaginalis*	50/654 (7.6%)	3/47
*M. hominis*	26/654 (4.0%)	0/26
*U. urealyticum*	16/654 (2.4%)	2/14
*U. parvum*	35/654 (5.4%)	5/30
*M. genitalium*	0/654	0/0
*Ca.* M. girerdii	0/654	0/0
*C. trachomatis*	21/654 (3.2%)	0/21
Uncultured bacteria	6/654 (0.9%)	1/5

Although samples from women were in the absolute minority in our collection (2.3%), we could confirm the presence of *T. vaginalis*, *U. parvum*, and *U. urealyticum* in 20% (3/15), 33% (5/15), and 13% (2/15) of samples, respectively, whereas in the male sample collection, *T. vaginalis*, *U. parvum*, and *U. urealyticum* were detected in 7.3% (47/639), 4.7% (30/639), and 2.5% (14/639) of samples.

### Co-infections detected in collected urine samples

Among the 50 *T. vaginalis*-positive samples, five were also positive for *M. hominis*, one for *M. hominis* and *C. trachomatis*, one for *U. urealyticum*, four for *U. parvum*, and one for “uncultured bacteria”. *Chlamydia trachomatis* co-infections with *M. hominis* were detected in two samples, and co-infections with *U. urealyticum* and *U. parvum* were observed in two samples each (Table 3). Among samples from females, *T. vaginalis* and *U. parvum* co-infections were detected in 3/15 (20%) and *T. vaginalis* with “uncultured bacteria” in 1/15 (6%).

**Table 3:** Prevalence (number of positive/total of tested population) of co-infections detected among asymptomatic soldiers (*n* = 654)

Pathogen	Prevalence (%)	Female/Male
*T. vaginalis* + *M. hominis*	5/654 (0.8%)	0/5
*T. vaginalis* + *M. hominis* + *C. trachomatis*	1/654 (0.1%)	0/1
*T. vaginalis* + *U. urealyticum*	1/654 (0.1%)	0/1
*T. vaginalis* + *U. parvum*	4/654 (5.4%)	3/1
*T. vaginalis* + uncultured bacteria	1/654 (0.1%)	1/0
*C. trachomatis* + *M. hominis*	2/654 (0.3%)	0/2
*C. trachomatis* + *U. urealyticum*	2/654 (0.3%)	0/2
*C. trachomatis* + *U. parvum*	2/654 (0.3%)	0/2

### Detection of *C. trachomatis* and serovar determination

We found *C. trachomatis* DNA in 21 of the collected urine samples (21/654, 3.2%), exclusively in males. Determination of serovars revealed that serovar E was the most common (12/21, 57%), followed by serovar D (7/21, 33.5%) and serovar F (2/21, 9.5%).

## Discussion

STIs are generally not a serious health risk for military personnel; however, because of the asymptomatic nature of some STIs, screening of sexually active military personnel is recommended [32]. In our cohort of 654 asymptomatic Austrian soldiers, *T. vaginalis* was the most commonly detected pathogen (7.6%), followed by *U. parvum*, *M. hominis*, *C. trachomatis*, and *U. urealyticum*, with prevalence of 5.4%, 4%, 3.2%, and 2.4%, respectively.

Infections with *T. vaginalis* are more frequent than infections with *C. trachomatis*, *Neisseria gonorrhoeae*, and *Treponema pallidum* combined; therefore, trichomoniasis represents the most common non-viral STD, with 156 million new cases estimated in 2016 [1]. In 2016, the global prevalence of trichomoniasis was estimated to be 0.6% for men and 5.3% for women [1]. Among young patients (both men and women) attending STI clinics in Sweden and Iceland, and women with vaginitis in Taiwan, *T. vaginalis* infections were rare [33–35], and the prevalence was not significantly different from that in the general population [1]. A considerable challenge for reliable estimations is the fact that up to 75% of men remain asymptomatic when infected with *T. vaginalis* [36]. In Korean military personnel, *T. vaginalis* was found as one of the important pathogens, with an incidence of 2.1%, 5.1%, and 5.7% in the 20–24 age group, 25–29 age group, and 40-and-over age group, respectively [37]. However, in an investigation of 400 cases of urethritis in male soldiers in the United States, no case of *T. vaginalis* infection was confirmed [38]. In a study on women with no gynaecologic complaints, the prevalence of *T. vaginalis* was 0.94% [39]. United States Army women tested for STIs revealed a percentage of *T. vaginalis* infection of 0.04% [40]. In our study, 20% (3/15) of the investigated women tested positive for *T. vaginalis*; however, the total sample size of 15 women is much too small for statistical evaluation. We should again emphasize that the samples used in our study were collected from a cohort of asymptomatic soldiers (no complaints reported), yet the overall prevalence of *T. vaginalis* (7.6%) was higher than the estimated prevalence of 0.3–1.3% in the general population and clinic attendees in Europe [39]. In a previous study performed in our laboratory in 2018, the prevalence of *T. vaginalis* among 338 asymptomatic soldiers was 1.13% (unpublished data), supporting the observation that there is a relatively higher prevalence of *T. vaginalis* in Austrian soldiers. On one hand, trichomoniasis may remain subclinical or may resolve due to the host immune system if left untreated; on the other hand, it can spread more widely when asymptomatic and increase the risk of acquisition and transmission of HIV, for example [41]. There are currently no guidelines available for optimal screening of asymptomatic individuals for *T. vaginalis*. The US Centers for Disease Control and Prevention (CDC) recommends screening for *T. vaginalis* infection in symptomatic women with vaginal discharge and in individuals at high risk of an STI (individuals with multiple sex partners, sex workers, drug addicts, or those who have a history of an STI) [42]. Our data indicate that at least in special settings, asymptomatic individuals should also be screened for the presence of *T. vaginalis* to mitigate the spread of this STI.

*Mycoplasma hominis* is associated with a very common flora alteration, bacterial vaginosis (BV) [43], and together with *Ureaplasma* spp. has been linked to a number of ailments, including infertility, low birth weight, stillbirth, preterm delivery, and PID [44, 45]. Colonization values of *M. hominis* on the mucosal surfaces of the cervix or vagina range between 20 and 30% around the world [46, 47]. In a Korean study on asymptomatic individuals, the prevalence of *Ureaplasma* spp. and *M. hominis* in women was 9.1% and 0.4%, respectively; 0.7% of them were positive for both *Ureaplasma* spp. and *M. hominis*, while, of the 956 tested specimens from males, only four (0.42%) were positive for *Ureaplasma* spp., and no *M. hominis* colonization was identified [48]. In the current study, *Ureaplasma* spp. were detected in 47% of the samples from female soldiers (no *M. hominis* was detected in this group), whereas among male soldiers only 6.8% were positive for *Ureaplasma* spp. and 4% for *M. hominis*. A study performed among asymptomatic young men in southern Thailand revealed that the prevalence of any urethral infectious agent was 15.9%, with *C. trachomatis* detected in 4%, *N. gonorrhoeae* in 0.2%, *U. urealyticum* in 10.9%, *M. genitalium* in 2.3%, and *M. hominis* in 1.3% [49]. In our asymptomatic cohort, the prevalence of *M. hominis*, *U. urealyticum*, and *U. parvum* was 4%, 2.4%, and 5.4% respectively, suggesting that *U. parvum* was the most abundant mollicute in this cohort. This is in line with findings from other studies, indicating a higher prevalence of *U. parvum* in the urogenital tract when compared with *U. urealyticum* [50].

In Poland, the prevalence of *Ureaplasma* spp. in women (14.4%) and men (3.9%) was higher than that of *M. hominis* in women (0.2%) and men (0.2%) with urogenital tract infection [51]. Hata et al. [52] reported the role and importance of *M. hominis* as a cause of neonatal meningitis, and Saadat et al. [53] noted that the high frequency of *M. hominis* in prostate cancer patients indicates a yet hidden role of these bacteria in the development of prostate cancer during chronic and asymptomatic colonization of the prostate. Moreover, it was shown that the prevalence of *M. hominis* in semen samples of infertile and fertile men varied from 3.2% to 18.2% and from 0.9% to 14.3%, respectively [54]. However, due to the presence of *M. hominis* and *Ureaplasma* spp. in both healthy women and patients with BV, it is still unclear whether *M. hominis* should be considered a pathogen at all. No evidence was found for *M. hominis* as a vaginal pathogen in adults [55], and the European Academy of Dermatology and Venereology recommended that patients (both asymptomatic and symptomatic) should not be screened for the presence of *M. hominis*. In a position statement from the European STI Guidelines Editorial Board, Horner et al. [56] did not recommend routine screening of asymptomatic men and women or routine testing of asymptomatic individuals for *M. hominis*, *U. urealyticum*, and *U. parvum*. In 2007, it was reported that the frequency of genital ureaplasmas and mycoplasmas detected in semen samples of infertile men in Tunisia was 19.2% and 15.8%, respectively [57]. Specifically, the frequency of *U. urealyticum* (15%) was higher than that of *M. hominis* (10.8%), *U. parvum* (4.2%), and *M. genitalium* (5%). They also detected mixed infections of mycoplasmas and ureaplasmas in 6.7% of semen samples. However, when they compared the semen parameters of infertile men with and without genital ureaplasmas and mycoplasmas, they could not show any significant differences. The conclusion was that it is not clinically relevant to screen mycoplasmas and ureaplasmas in routine semen analysis. Further studies are needed to evaluate the roles of these Mollicutes in regard to symptoms, as well as sequelae in asymptomatic individuals.

In our cohort, we did not detect *Ca.* M. girerdii, despite the high number of *T. vaginalis*-positive samples. Even though *Ca.* M. girerdii has been previously detected and defined as an emerging *Mycoplasma* spp. associated with *T. vaginalis* infection, there is no supporting evidence of an endosymbiotic relationship between these two organisms [21, 58]. It has been reported that *Ca.* M. girerdii as well as other uncultivable *Mycoplasma* species can only be directly detected in association with *T. vaginalis* in vaginal discharge and not in pure *T. vaginalis* cultures [58, 59]. We used urine samples for the detection of parasites and bacteria, which might explain why we could not detect *Ca.* M. girerdii.

Also, *M. genitalium* was not detected in our study cohort. The prevalence of *M. genitalium* varies depending on the country and the patient population. *Mycoplasma genitalium* has been detected in a range from 1% in the general population to 42% in African men with NGU [60]. In the general population, the prevalence of *M. genitalium* is estimated to be between 1% and 2% [61, 62] while in patients attending sexual health clinics, it ranges from 3.3% to 38% [63–66]. In a study performed on 2505 cases of male urethritis, using first-void urine, the positivity rate for *M. genitalium* was 23% [66]. Taylor-Robinson and Jensen in 2011 demonstrated *M. genitalium* in 15–25% of men with symptomatic NGU, and in 5–10% of those without NGU [67], thus indicating *M. genitalium* as causative agent for NGU. Andersen et al. [68] estimated the prevalence of *M. genitalium* in the general population in Denmark to be 2.3% in women and 1.1% in men. For men with urethritis and women with cervicitis, *M. genitalium* prevalence was 19% and 11%, respectively, in that study. In consideration of these data, it was very surprising that we did not detect *M. genitalium* in our samples, despite using two different methods.

In contrast to gonorrhoea, more than 90% of *C. trachomatis* infections in men are asymptomatic, whereas in women, 70–80% do not show symptoms [69]. Chlamydiasis remains the most frequently diagnosed venereal disease in populations of soldiers; however, we detected *C. trachomatis* in only 3.2% of the male soldiers and in none of the female soldiers. A study on Korean soldiers affected by urethritis revealed that *C. trachomatis* was the most frequently identified causative agent (36.6%), followed by *U. urealyticum* (24%), *M. genitalium* (21.5%), *N. gonorrhoeae* (19%), *M. hominis* (6.1%), and *T. vaginalis* (0.2%) [70]. In a military cohort in Poland [32], a very low proportion (0.8%) of *C. trachomatis* infection was observed, even though 40% of the soldiers reported sexual contact with different partners within 12 months before evaluation. Data available for French military personnel showed that *C. trachomatis* was the predominating infectious agent [71]. These data indicate that the incidence of *C. trachomatis* varies to a great extent, even when investigating similar target groups (soldiers). Trei et al. [72] reported that men diagnosed with chlamydiasis showed a slight increase in the risk of prostatitis but also a four times higher risk of epididymitis. Lesiak-Markowicz et al. showed in 2019 [73] that serovar E was the most common type (50.1%) in Austrian patients (both genders were tested, with a majority of female patients). Our results confirm these findings, indicating that serovar E is also the most common one (57%) in asymptomatic male soldiers, followed by serovar F (33%) and serovar D (10%). Generally, serovars D, E, and F are known to be the most prevalent worldwide [74].

## Conclusions

Our study plays an important role as it provides data on STIs in a predominantly male cohort, which is rare, as most of the available information on STIs comes from fertility clinics (mainly women) or from symptomatic patients. In samples from asymptomatic patients, we detected the presence of *T. vaginalis*, *M. hominis*, *U. urealyticum*, *U. parvum*, and *C. trachomatis*. We believe that our research can be used to consider whether asymptomatic samples should be screened for *M. hominis*, *U. ueralyticum*, and *U. parvum*.

## Limitations

This study has several limitations. Firstly, mixed samples were used, containing first-void and midstream urine (standard urine sample). Secondly, due to the retrospective character of the study, only information on the gender of the soldiers was available, and the small number of samples from female soldiers also limits the statistical evaluation.

## Data Availability

All data generated or analysed during this study are included in this published article.

## Abbreviations

WHO: World Health Organization
STI: Sexually transmitted infection
BV: Bacterial vaginosis
NGU: Non-gonococcal urethritis
PID: Pelvic inflammatory disease
*T. vaginalis*: *Trichomonas vaginalis*
*M. hominis*: *Mycoplasma hominis*
*M. genitalium*: *Mycoplasma genitalium*
*Ca.* M. girerdii: *Candidatus* Mycoplasma girerdii
*U. urealyticum*: *Ureaplasma urealyticum*
*U. parvum*: *Ureaplasma parvum*
ISPTM: Institute of Specific Prophylaxis and Tropical Medicine
MOMP: Major outer membrane protein
CDC: Centers for Disease Control and Prevention
